# Electrodermal Response to Mirror Exposure in Relation to Subjective Emotional Responses, Emotional Competences and Affectivity in Adolescent Girls With Restrictive Anorexia and Healthy Controls

**DOI:** 10.3389/fpsyg.2021.673597

**Published:** 2021-09-10

**Authors:** Terézia Knejzlíková, Miroslav Světlák, Tatiana Malatincová, Robert Roman, Jan Chládek, Jana Najmanová, Pavel Theiner, Pavla Linhartová, Tomáš Kašpárek

**Affiliations:** ^1^Department of Psychiatry, Faculty of Medicine, Masaryk University, Brno, Czechia; ^2^Institute of Psychology and Psychosomatics, Faculty of Medicine, Masaryk University, Brno, Czechia; ^3^Institute of Scientific Instruments of the CAS, Brno, Czechia

**Keywords:** mirror exposure, anorexia nervosa, skin conductance, emotion awareness, interoceptive awareness

## Abstract

**Objective:** Body image disturbances and the attendant negative emotions are two of the major clinical symptoms of eating disorders. The objective of the present experimental study was to shed more light on the degree of association or dissociation between the physiological and emotional response to mirror exposure in patients with restrictive mental anorexia, and on the relationships between the physiological response and characteristics connected with emotional processing.

**Materials and Methods:** Thirty adolescent girls with the restrictive type of anorexia and thirty matched healthy controls underwent bilateral measurement of skin conductance (SC) during rest, neutral stimulus exposure, and mirror exposure, and completed a set of measures focused on emotion regulation competencies, affectivity, and eating disorder pathology.

**Results:** Compared to healthy controls, girls with restrictive anorexia rated mirror exposure as a subjectively more distressful experience. Differences in skin conductance response (SCR) were not significant; however, variance in SCR was substantially greater in the group of anorexia patients as compared to healthy controls. The overall skin conductance level (SCL) was lower in anorexia patients. Increase in SCR during mirror exposure, as opposed to exposure to neutral stimuli, was positively related to the tendency to experience negative emotions, interoceptive sensitivity, body dissatisfaction and suppression, but not to other symptoms of eating pathology or emotional awareness. A *post hoc* analysis suggested that physiological reactivity might be associated with interoceptive sensitivity to mirror exposure especially in anorectic patients.

**Conclusion:** The study seems to demonstrate some degree of dissociation between psychophysiological reactivity and subjective response to body exposure in patients with restrictive anorexia. Factors affecting differences in psychophysiological responsiveness to body exposure in anorectic patients require further exploration.

## Introduction

Disturbances in body shape perception are one of major clinical symptoms in women and girls suffering from eating disorders (EDs), especially bulimia nervosa (BN) and anorexia nervosa (AN; Bruch, 1962). In these disorders, body is the primary source of negative emotions (Cash and Pruzinsky, 2004; Keizer et al., 2011; Svaldi et al., 2016), which cannot be adaptively regulated (Kaye et al., 2004; Oldershaw et al., 2015). These issues are also considered principal etiological factors of EDs, contributing both to the precipitation and the perpetuation of EDs (Killen et al., 1996; Stice and Whitenton, 2002; Espeset et al., 2012).

A recent systematic review (Griffen et al., 2018) showed that looking in the mirror can induce distress and negative emotions, especially in women with negative body image and eating pathology. The act of viewing oneself in a mirror triggers a complex psychological response, affected greatly by the level of satisfaction/dissatisfaction with one’s body (Glashouwer et al., 2016), emotional valence attached to one’s body, cognitive self-regulation skills that help manage the elicited negative emotions (Crino et al., 2019), and current mood quality (Svaldi et al., 2016). This response drives individuals with EDs to keep checking their body frequently, engage in body avoidance behavior (e.g., covering mirrors), or switch between these two strategies (Shafran et al., 2004). Variability (flexibility vs. rigidity) in these behavioral responses appears to be significantly influenced by the emotion regulation strategies employed (Brockmeyer et al., 2014), as well as etiological heterogeneity of ED cases (Kaye et al., 2009). Recent findings seem to converge on the idea that the central function of EDs like AN or BN could be understood as an attempt to control unwanted or dysregulated emotions (Bydlowski et al., 2005; Lavender et al., 2015). In this view, desire to control and change one’s body represents an attempt to control dysregulated emotions without being aware of their primary content (Kearney-Cooke and Tieger, 2015).

In line with the above, multiple recent studies found impairments in various emotional competences in EDs (Lavender et al., 2015). One of them, first described by Bruch (1962) as “marked deficiencies in identifying emotional states” (p. 254) in women with EDs and investigated extensively ever since, is diminished awareness of one’s emotional states and the corresponding interoceptive component. *Emotional awareness* can be defined as the extent to which one can identify and describe one’s own emotions (Moon and Berenbaum, 2009). It represents the ability to consciously perceive and be aware of emotional states as well as the capability to match emotional experiences with appropriate semantic categories. Emotional awareness is closely linked to *interoception*, i.e., the sensing of internal bodily changes (Garfinkel et al., 2015). According to the somatic marker hypothesis, conscious feelings originate from the perception of bodily sensations and their dynamic changes (Damasio, 1999). In this perspective, the ability to consciously perceive and be aware of somatic components of affective states is considered essential for an authentic experience of self (Dolhanty and Greenberg, 2007) and a prerequisite for adaptive emotion regulation (Gross and Jazaieri, 2014). The link between restriction and decreased emotional awareness and interoceptive sensitivity in restrictive AN patients—demonstrated, for example, by studies documenting associations between caloric deprivation and downregulation of physiological arousal (Miller et al., 2003) and elevated pain threshold (Papezová et al., 2005)—might be a reflection of a downward spiral involving a bidirectional link between emotional and behavioral dysregulation (Van Strien et al., 2005; Selby et al., 2008). It can be argued that the permanently decreased emotional awareness and interoceptive sensitivity are actually outcomes of non-adaptive but—from the point of view of women with restrictive AN—successful emotion regulation. The originally effortful and conscious strategies to regulate unwanted emotions (i.e., restriction in women with restrictive AN) gradually transform into automatic/unconscious emotion regulation strategies.

In the past few decades, mirror exposure has been used with ED patients as a unique, easily accessible, transdiagnostic treatment and experimental tool with high ecological validity (Servián-Franco et al., 2015). “Mirror exposure” refers to viewing oneself in a mirror with or without specific guidance for a predetermined amount of time. Moreno-Domínguez et al. (2012) distinguish between “pure” mirror exposure and “guided” mirror exposure. In pure mirror exposure, subjects simply view their whole bodies with or without commenting on the elicited emotions and cognitions, without any further directions from the experimenter or therapist. In contrast, in guided mirror exposure, subjects are directed to focus on specific parts of their bodies and can be asked to describe the emerging thoughts and feelings in a more controlled manner (Moreno-Domínguez et al., 2012; Díaz-Ferrer et al., 2017). As a research tool, mirror exposure has been used to study not only the quality and regulation of emotional responses to one’s own body (Vocks et al., 2007; Trentowska et al., 2013; Servián-Franco et al., 2015), but also the long-term process of body acceptance or normalization of body image resulting from repeated body exposure (Key et al., 2002; Delinsky and Wilson, 2006; Trentowska et al., 2014).

Since body dissatisfaction is a major symptom of EDs like AN and BN, it seems logical that body exposure *via* a mirror would be a significantly more stressful experience for individuals suffering from these disorders than it is to healthy individuals. However, research findings seem to paint a more complex picture. First, it appears that subjective emotional and cognitive responses to mirror exposure in women diagnosed with EDs do not have to be substantially different from those in body-dissatisfied women who otherwise do not meet the diagnostic criteria for an ED (Cooper and Fairburn, 1992; Tuschen-Caffier et al., 2003). Second, data regarding subjective indicators of distress arising in response to mirror exposure appear to be surprisingly at odds with data regarding physiological indicators. For example, Vocks et al. (2007) documented that while women with EDs tended to initially report higher levels of negative emotions and cognitions in response to body exposure *via* a mirror as compared to healthy controls, there were no group differences in psychophysiological response as indexed by heart rate, skin conductance, or saliva cortisol levels. Interestingly, when mirror exposure was extended over a period of 40 min, self-reported negative emotions and cognitions in the ED group gradually decreased, while the psychophysiological parameters remained unchanged. More recently, Servián-Franco et al. (2015) found that, compared to low body-dissatisfied women, highly body-dissatisfied women showed a seemingly paradoxical reduction in physiological response after a 5-min guided mirror exposure, although their self-reported levels of negative emotions and cognitions were higher. Moreover, physiological indicators were only associated with subjective indicators in the low body-dissatisfied group.

While both of the studies cited above indicate some level of physiological desensitization, it is not clear whether this phenomenon applies to all body-disatisfied individuals or is only associated with specific subtypes of emotion dysregulation and ED-related pathology. Recent research in this area does not differentiate between different subtypes of AN; however, there are consistent reports of permanently decreased activation of the autonomic nervous system in AN, especially as indexed by skin conductivity parameters (Vocks et al., 2007; Gorini et al., 2010; Pruneti et al., 2010; Morgan et al., 2014). This reduced autonomic reactivity in AN patients can be explained by changes in sympathetic reactivity induced by metabolic, emotional and cognitive abnormalities in the restrictive type of AN (Palomba et al., 2017) and might be at least partly responsible for the seemingly paradoxical findings on physiological responsiveness to mirror exposure in body-dissatisfied women. The muted psychophysiological reactivity may then, in turn, be a significant factor contributing to the decreased body and emotional awareness and the posited dissociation between the cognitive, emotional, and physiological levels of experience in patients with restrictive AN (Zonnevylle-Bender et al., 2005; Nandrino et al., 2012).

In the present study, we focused specifically on the differences between emotional responses to mirror exposure in adolescent female patients with restrictive AN and paired healthy controls, and in psychological characteristics related to emotional responsiveness, emotional and interoceptive awareness and emotion regulation, which might modulate these emotional responses. Specifically, we addressed the questions of whether subjective emotional responses to mirror exposure in adolescent girls suffering from restrictive AN differ from those in healthy controls (HCs), and whether these expected differences in subjective assessment are accompanied by corresponding changes in physiological indicators of arousal, namely skin conductance parameters (SC), or electrodermal activity (EDA). We hypothesized that girls with AN perceive mirror exposure as a more upsetting experience, rating it as more arousing, more negative and less controllable as compared to paired HCs. We also hypothesized that AN patients show higher EDA, as indexed by changes in phasic SC, in response to mirror exposure, as compared to HCs. This difference was not expected after exposure to a neutral stimulus, which was expected to elicit a generally weaker response than mirror exposure. Based on previous findings documenting a general hypoactivation of the autonomic nervous system in AN (Vocks et al., 2007; Servián-Franco et al., 2015; Glashouwer et al., 2016; Svaldi et al., 2016; Palomba et al., 2017; Crino et al., 2019), we also expected AN patients to show generally lower levels of EDA, indexed by tonic SC, as compared to HCs across all conditions (rest, exposure to neutral stimuli, and mirror exposure).

Regarding individual-difference variables, we expected that increase in EDA, especially in phasic SC, in response to mirror exposure, would be positively related to the level of restriction as indexed by BMI, drive for thinness, body dissatisfaction, the overall level of negative emotional experiences, and employment of suppression as an emotion regulation strategy, and negatively related to the overall level of positive emotional experiences, emotional and interoceptive awareness, and the employment of cognitive reappraisal as emotion regulation strategy. Finally, logically following from both of the above sets of hypotheses, we expected AN patients to score significantly higher in individual-difference variables positively associated with an increase in SC after mirror exposure, and lower in variables negatively associated with an increase in SC after mirror exposure, as compared to HCs.

## Materials and Methods

### Participants

Thirty adolescent girls (Mean age = 14.9 ± 1.31) with the restrictive type of AN were recruited from the Department of Psychiatry, Child and Adolescent Inpatient Unit of the University Hospital. To eliminate the impact of short-term and long-term habituation during mirror exposure, the study was conducted on patients at the start of their hospitalization at the children’s department. In cases of severe malnutrition caused by the ED, patients are first treated and nourished at intensive care units before transferring to the Department of Psychiatry. Thus, our sample contained no participants in acute phases of malnutrition. The diagnosis was established using the Structured Clinical Interview for DSM-5 (American Psychiatric Association, 2013) conducted by a board-certified psychiatrist. The originally recruited group consisted of 35 patients; however, five patients were excluded in the assessment process, two because they met the DSM-5 criteria for the purging type of AN, and three because there was an episode of bulimia nervosa in their anamnesis. The final AN group thus included 30 patients with an average duration of hospitalization of 51 days (included days spent at the hospital after the experiment, which was conducted with every participant within the first week of her hospitalization). Twenty-two patients were hospitalized for the first time, 7 for the second time, and one for the third time. The average BMI was 16.9 ± 3.73 kg/m^2^. Using the eight age-adjusted centile groups for the Czechia population of children and adolescents (Bláha et al., 2005), 19 girls were classified as severely underweight (centiles < 3) and 6 as mildly underweight (centiles 3 to 10). There were four “atypical” patients in our clinical group who showed normal weight (BMI = 21.15 and 24.09 kg/m^2^) or even overweight (BMI = 27.42 and 27.70 kg/m^2^) at the time of the study, but were nevertheless included in sample, as they were hospitalized due to a long history of restrictive anorexia and were facing significant restriction of diet and drinking at the time of hospitalization. There were no officially diagnosed psychiatric comorbidities; however, the patients’ clinical record files frequently cited symptoms of depression, anxiety and obsessive-compulsive behavior.

For the control group (HCs), thirty girls (Mean age = 15.7 ± 1.09) were recruited among students at primary and secondary schools. An official invitation was advertised by collaborating head-teachers, in which eligible female students were instructed to apply directly to the research team if interested. No financial reward was offered for participation, but the students were allowed to take a day off school to attend the experimental session. The group was matched to the AN group on age/developmental stage/school year and educational level; however, average age was somewhat higher in the control group [*t*(60) = 2.35, *p* < 0.05]. The principal exclusion criterion was EDs in medical history (past or ongoing). The absence of EDs was further validated by anamnestic interview conducted by a clinical psychologist. The mean BMI for the HC group was 21.1 ± 2.6 kg/m^2^. Most girls showed normal weight (centiles 10 to 90, *n* = 21). One girl was classified as severely underweight (BMI = 16.9 kg/m^2^) but did not show any other symptoms of EDs.

General exclusion criteria for both groups were personality disorders, psychotic disorders, diseases that affect physiological reactivity (diabetes mellitus, thyroid disorder, coronary artery disease, etc.), and medication influencing the activity of the sympathetic nervous system and adrenocortical response to stress (Vocks et al., 2004). All participants were right-handed according to the Waterloo Handedness Questionnaire (Elias et al., 1998). All participants and their parents signed the informed consent form.

### Psychological Measures

#### Eating Pathology

Symptoms of eating pathology were assessed by the Eating Disorder Inventory (EDI; Garner et al., 1983) translated into Czechia for the purpose of the present study. The original inventory consists of 64 items rated on a 6-point scale ranging from “always” to “never,” capturing attitudes and behavior associated with core features of eating disorders (Garner and Barry, 2001). Out of the eight EDI subscales, only three were used in the present study: Dive for Thinness (DFT), Bulimia (B), and Body Dissatisfaction (BD). The DFT scale consists of seven items that focus on extreme desire to be thinner, intense fear about weight, and concern with dieting. The B scale consists of seven items and focuses on overeating and eating in response to being upset. The BD scale consists of nine items, focusing on discontent with one’s body shape and specific parts of one’s own body. All three scales showed good internal consistency (α = 0.73 to 0.87). The Interoceptive Awareness (IA) was not included in the analysis due to psychometric problems (i.e., several items showing near-zero correlations with the rest of the scale). Instead, interoceptive awareness was measured by a different instrument (see below).

#### Emotional Awareness

The level of emotional awareness was assessed by the Level of Emotional Awareness Scale for Children (LEAS-C, Bajgar et al., 2005). LEAS is a performance-based tool designed to measure individual differences in the capacity to experience feelings in a differentiated and complex way and to detect such feelings in others (Lane et al., 1990). It consists of 12 descriptions of interpersonal situations involving two people, one introduced as the respondent and the other as another person. The respondents are asked to answer two questions: *How would you feel in this situation?* and *How would the other person feel in this situation?* The complexity of emotional response is scored using the word list included in the manual (Barchard et al., 2010). Responses are assigned between 1 and 4 points based on the cognitive-developmental theory proposed by Lane and Schwartz (1987). The Czechia adaptation of LEAS showed high interrater reliability (Světlák et al., 2017). In the present study, we only used the self-awareness score (LEAS-self), which is calculated as the total score for all 12 responses to the first question. The score showed acceptable internal consistency in our sample (α = 0.75).

#### Interoceptive Awareness

Interoceptive awareness was assessed by two subscales of the Body Perception Questionnaire (BPQ; Porges, 1993). In the Awareness subscale (BPQ: AW), consisting of 45 items, respondents are asked to rate how often they are aware of their own bodily processes and signs of different kinds. The Autonomic Nervous System Reactivity subscale (BPQ: ANS) consists of 27 items measuring responses of the respondent’s autonomous nervous system during an emotional event. All items are rated on a 5-point scale from “Never” to “Always.” The Czechia version used in the present study was provided by Bernátová and Světlák (2017). Internal consistencies of both subscales were high (α = 0.96 and 0.89, respectively).

#### Emotional Experiences

The Subjective Emotional Balance Questionnaire (SEBQ; Kožený, 1993) assesses the frequency of positive and negative emotional experiences. The questionnaire consists of the Positive Emotional Experiences subscale (e.g., “I felt calm and relaxed”; “I was cheerful”) and the Negative Emotional Experiences subscale (e.g., “I was in a bad mood”; “I was unhappy”), with 18 items per subscale. The items are rated on a scale from 1 (“almost never”) to 5 (“very often”). Internal consistencies of both subscales were high in the present sample (α = 0.95 and 0.93, respectively).

#### Emotion Regulation Strategies

The Emotional Regulation Questionnaire (ERQ; Gross and John, 2003) measures individual differences in the habitual use of two types of emotion regulation strategies: cognitive reappraisal and suppression of emotions. The ERQ consists of 10 items rated from 1 (“strongly disagree”) to 7 (“strongly agree”). The Czechia version used in present study has shown good psychometric properties (Marsova, 2016).

#### Current Emotional Response

**FIGURE 1:**
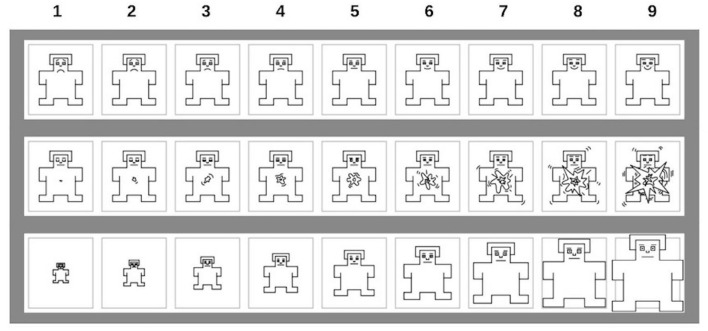
The Self-Assessment Manikin graphical scale (SAM).

The Self-Assessment Manikin (SAM; Bradley and Lang, 1994; Figure 1) is a tool commonly used for the assessment of three basic dimensions—valence, arousal, and dominance—of ongoing emotional states. Each dimension is rated by the respondent on a 9-point bipolar graphical scale. Emotional valence (first row in Figure 1) is rated on a scale from extremely unpleasant (unhappy manikin on the left) to extremely pleasant (happy manikin on the right), arousal (second row in Figure 1) is rated on a scale from calm (relaxed manikin on the left) to extremely excited (aroused manikin on the right), and dominance (third row in Figure 1), describing the level of subjectively perceived sense of control over the emotion evoked by the stimulus, is rated on a scale from complete loss of control (manikin on the left) to full control (manikin on the right). The subject is instructed to rate his/her immediate subjective feelings elicited by the presented stimulus (i.e., body reflection in the mirror in the present study) on the three afore-mentioned dimensions. In our study, the emotional valence scale was reversed for better interpretability with respect to the other SAM scales (i.e., higher values indicate negative valence).

### Procedure

Both groups completed the questionnaires with a certified clinical psychologist’s assistance one day before the experiment. The experiment itself was conducted during a standardized period of time for every girl, always in the morning between 8:30 and 10:30 a.m. The participants were instructed not to drink alcohol, smoke, or use any drugs the night before the experiment and were asked to get enough sleep and have breakfast while abstaining from coffee or energy drinks. All AN participants completed the experiment within the first week of their hospitalization.

The measurements were performed in a quiet room with a temperature of ca. 23°C. Two pairs of Ag/AgCl electrodes (8 mm active area diameter) filled with electroconductive paste were used, attached to the medial phalanges of the index and the middle finger of each hand. During the experiment, the subject sat in a comfortable chair, dressed in standardized clothes (white leggings and a white tank top). The experimenter was in another room, communicating with the participant using a video camera and microphone. All experimental instructions were standardized and pre-recorded, spoken by a calm male voice and played to the subject from speakers located in the experimental room.

**FIGURE 2:**
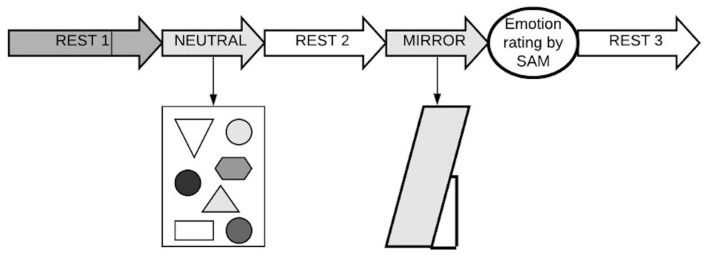
The experimental design in the present study. REST 1 = last 1 min from a 3-min rest period; NEUTRAL = 30 s neutral condition (“vanilla baseline”); REST 2 = 1-min rest after neutral condition, MIRROR = 30 s mirror exposure, SAM, Self-Assessment Manikin scale, REST 3, 1-min rest after mirror exposure period.

The course of the experiment is outlined in Figure 2. After an initial 5-min rest, the subject was given the instruction: *“Please, close your eyes and rest as usual.”* The first EDA measurements were taken during these additional 3 min of resting with eyes closed, the last minute of which was set as the baseline (REST 1). After 3 min, the subject was invited to open her eyes (*“Please, open your eyes and look at the picture in front of you”*) to view geometric shapes in shades of gray (Jennings et al., 2007), which were printed on a large sheet of paper entirely covering a mirror that stood in front of the subject. This was the neutral condition of the experiment (NEUTRAL), which lasted 30 s. Then the subject was invited to close her eyes and rest again (“*Please, close your eyes and rest as usual”*; REST 2). After 1 min, the subject was invited to open her eyes again to view her own reflection in the mirror (“*Please, open your eyes and look in the mirror in front of you”*; MIRROR), which, in the meantime, was uncovered by the experimenter. We used pure mirror exposure without any specific instructions regarding emotion regulation or body scanning (Moreno-Domínguez et al., 2012). Whether the subjects were actually watching themselves in the mirror was checked by the examiner through the camera aimed at the subjects from the side. The MIRROR condition took 30 s and was followed by the subject’s oral assessment of her ongoing emotional state using the Self-Assessment Manikin scale, which was printed and presented in a visible place in the experimental room. All participants were introduced to the SAM before the experiment to ensure they interpreted the graphical scales correctly. After rating her current emotional state, the subject was once again asked to rest with her closed eyes for another minute (REST 3). Data from this last resting period were not included in the analysis.

### Skin Conductance Measures and Data Analysis

Skin conductance (SC) was recorded with the Psychlab data acquisition system (Contact Precision Instruments) with a sampling rate of 1,000 Hz. Ledalab software V3.4.9 was used for the SC data analysis. The raw data were downsampled to 20 Hz, corrected for artifacts, and decomposed into tonic skin conductance level (SCL) and phasic skin conductance response (SCR) components using the continuous decomposition analysis (Benedek and Kaernbach, 2010). The SC was measured bilaterally. Since the Spearman correlations between data obtained from the left hand and the right hand during the baseline condition were very strong for both SCL (*r* = 0.88, *p* < 0.001) and SCR (*r* = 0.97 *p* < 0.001), all other analyses were performed with data obtained from the dominant hand (i.e., right hand in all participants).

### Statistical Analysis

Skin conductance data, which were severely positively skewed, were transformed prior to data analysis. SCR data were transformed using the *y* = *ln(0.00001* + *x)* function. With SCL data, which were less severely skewed, square-root transformation was used. After transformation, there were no significant deviations from normal distribution, nor were there any significant outliers in the data set.

Data analyses were conducted using IBM SPSS Statistics, version 25, and R, version 4.0.3. To test the major hypotheses regarding the effect of mirror exposure on physiological and subjective psychological indicators of distress, we conducted a series of mixed-design 2 × 4 ANOVAs. Where the assumption of homogeneity of variances was violated, we used the bwtrim function of the WRS2 package for R (Mair and Wilcox, 2020) to perform a robust ANOVA based on trimmed means. Group differences in psychological variables and BMI were tested using between-subject *t*-tests with bootstrapped *SE*s. Pearson correlation coefficients were also computed with bootstrapped *SE*s. Spearman correlation coefficients were used for the BMI centile band. A supplementary principal component analysis was conducted to explore common variance between self-report variables included in the study.

## Results

### Skin Conductance Differences Between Groups and Conditions

**FIGURE 3:**
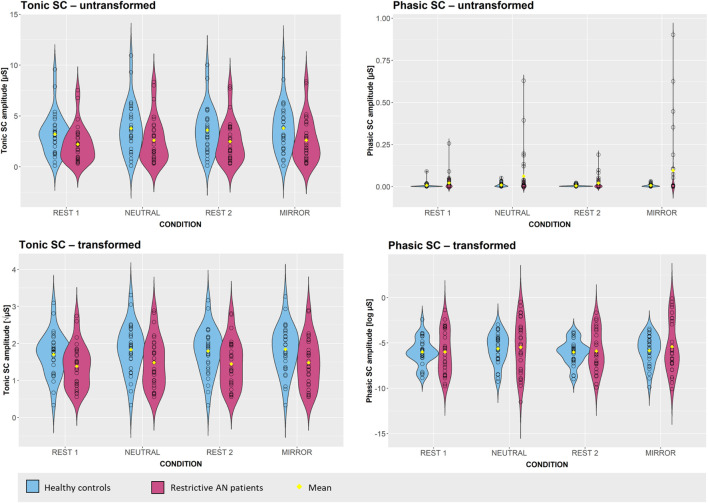
Violin plots of electrodermal responses in AN patients and matched healthy controls across conditions.

Table 1 shows the descriptive statistics of SC parameters before and after transformation. The data are also graphically represented in Figure 3 to show the differences between the groups and conditions. The values clearly indicate that, across all conditions, the AN group showed considerably greater variance, especially in SCR, as compared to HCs. Correlations across conditions in the entire sample were generally very strong for SCL, with *r*s ranging between 0.956 and 0.995 in untransformed data, and 0.957 and 0.995 in transformed data. For SCR, the correlations were mostly stronger and much less variable in transformed data (*r*s ranging from 0.782 to 0.901) as compared to untransformed data (*r*s ranging from 0.402 to 0.947), which was apparently the result of severe skew and presence of extremely high outlier values in the untransformed data. Table 1 also shows that between-subject variance in SCR was generally higher in the stimulus exposure conditions and was particularly high in the AN group.

**TABLE 1:** Descriptive statistics for skin conductance data by group and condition.

			Untransformed data					Transformed data	
Variable	Group	Condition	M	SD	MD	Min	Max	M	SD
Tonic skin conductance level (SCL)	AN	REST 1	2.22	1.78	1.95	0.31	7.53	1.38	0.57
		NEUTRAL	2.62	2.21	1.96	0.31	8.34	1.48	0.66
		REST 2	2.49	2.05	1.87	0.31	7.90	1.45	0.64
		MIRROR	2.61	2.17	2.62	0.31	8.39	1.48	0.66
	HC	REST 1	3.22	2.04	3.15	0.11	9.58	1.70	0.58
		NEUTRAL	3.78	2.47	3.58	0.11	10.95	1.83	0.66
		REST 2	3.61	2.27	3.48	0.11	10.02	1.79	0.63
		MIRROR	3.79	2.38	3.50	0.11	10.69	1.84	0.65
Phasic skin conductance response (SCR; for untransformed data, displayed values represent results multiplied by 10^4^)	AN	REST 1	258	492	24	0	2,560	–5.99	2.39
		NEUTRAL	620	1,371	46	0	6,290	–5.47	2.94
		REST 2	204	407	19	0	1,900	–5.89	2.27
		MIRROR	962	2,130	22	0	9,020	–5.42	2.86
	HC	REST 1	76	166	24	1	900	–6.00	1.53
		NEUTRAL	94	121	43	1	509	–5.63	1.69
		REST 2	51	63	29	1	226	–6.01	1.36
		MIRROR	71	84	37	0	319	–5.84	1.64

*REST 1, last 1-min from 3-min rest; NEUTRAL, neutral exposure condition with geometric shapes; REST 2, rest between neutral condition and mirror exposure; MIRROR, experimental condition with mirror exposure; AN, patients with anorexia nervosa; HC, health control group.*

Two two-way 2 × 4 mixed-design analyses of variance were conducted to test the effects of group (HC vs. AN) and condition (REST 1 vs. NEUTRAL vs. REST 2 vs. MIRROR) on the transformed values of SCL and SCR, respectively. For SCL, Mauchly’s test revealed a significant violation of the assumption of sphericity [χ^2^(5) = 102.86, *p* < 0.001]. Degrees of freedom for the main within-subject effect and the interaction effect were therefore corrected using the Greenhouse-Geisser estimate of sphericity (ε = 0.57). The two-way 2 × 4 mixed-design revealed a significant main effect of group [*F*(1, 58) = 4.66, *p* = 0.03, η_*p*_^2^ = 0.07], with anorexia patients showing lower levels of SCL compared to controls across all conditions (see Table 1 and Figure 3). The main effect of condition on SCL was also significant [*F*(1.79, 99.07) = 14.32, *p* < 0.001, η_*p*_^2^ = 0.20]. Bonferroni *post hoc* comparisons revealed that levels of SCL in the whole sample were significantly higher in both exposure conditions (NEUTRAL and MIRROR, both *p* < 0.001) as compared to REST 1. SCL measured during REST 2 was significantly lower than SCL in MIRROR (*p* = 0.009) and was also lower compared to SCL measured in NEUTRAL, but this difference failed to reach significance (*p* = 0.06). SCL measured during REST 1 was non-significantly lower than SCL in REST 2 (*p* = 0.05). The difference between SCL in NEUTRAL and MIRROR was close to zero (*p* = 1.00). The interaction effect of group × condition on SCL was negligible and non-significant [*F*(1.79, 99.07) = 0.51, *p* = 0.58, η_*p*_^2^ < 0.01].

For transformed SCR, no violations of the sphericity assumption were detected; however, Levene’s test indicated violations of the assumption of homogeneity of variances across all conditions (all *p*s < 0.02). For this reason, apart from the standard mixed-design ANOVA, we conducted a supplementary robust mixed-design ANOVA based on trimmed means. We report the results of both analyses here, bearing in mind that using trimmed means was likely to change the results substantially due to the asymmetry of outlier distribution in the two groups in our study (see Figure 3). The standard two-way 2 × 4 mixed-design ANOVA revealed no significant effect of group on transformed SCR [*F*(1, 58) = 0.14, *p* = 0.71, η_*p*_^2^ < 0.01], but there was a significant main effect of condition [*F*(3, 174) = 4.42, *p* < 0.01, η_*p*_^2^ = 0.07]. The interaction between group and condition was not significant [*F*(3, 174) = 0.52, *p* = 0.67, η_*p*_^2^ < 0.01]. These results were confirmed by the robust ANOVA with a trim level of 10% [Main effect of group: *F*(1, 34.24) = 0.01, *p* = 0.92; Man effect of condition: *F*(3, 37.36) = 4.37, *p* < 0.01; Interaction effect of group × condition: *F*(3, 37.36) = 0.11, *p* = 0.95] as well as a trim level of 20% [Main effect of group: *F*(1, 22.23) = 0.01, *p* = 0.94; Man effect of condition: *F*(3, 27.57) = 5.05, *p* < 0.01; Interaction effect of group × condition: *F*(3, 27.57) = 0.11, *p* = 0.98]. For the main effect of condition, Bonferroni *post hoc* tests indicated that SCR was significantly lower during REST 2 as compared to both NEUTRAL (*p* = 0.02) and MIRROR (*p* = 0.05). Differences in SCR between REST 1 and the two exposure conditions were of similar size but non-significant. Differences between the two exposure conditions, as well as differences between the two resting conditions, were negligible.

### Psychological Measures

#### Group Differences in Psychological Variables and Self-Assessed Emotional Responses to the Mirror Exposure

Differences between the AN and HC groups in psychological variables and BMI were tested using independent sample *t*-tests. Levene’s test revealed no significant differences in variances between the two groups. Results of the *t*-tests are displayed in Table 2. As expected, the AN group scored significantly higher in all three indicators of subjective emotional state after mirror exposure, with effect sizes ranging from medium to large. The AN group also scored significantly higher in the Body Dissatisfaction EDI subscale and the Negative Affect subscale of SEBQ and showed lower BMI, lower Positive Affect SEBQ scores and lower LEAS-self emotional awareness scores. The AN group also scored higher in the EDI Drive for Thinness and Bulimia subscales, but these differences were non-significant. Contrary to our expectations, the AN group scored higher, rather than lower, in interoceptive sensitivity represented by BPQ scores, but this difference was not significant either. Differences between the two groups in emotion regulation strategies (ERQ) were negligible.

**TABLE 2:** Descriptive statistics and mean differences in SAM, EDI, LEAS-C, BPQ, SEBQ, and ERQ scores and BMI between AN patients and matched HCs (no Type I error correction applied).

	HC		AN					Hedge’s *g*
		
	*n*	*m (sd)*	*n*	*m (sd)*	*T*	*df*	*p* ^*a*^	
**SAM: Valence**	**30**	**4.33 (1.21)**	**29**	**5.31 (1.73)**	−**2.51**	**57**	**0.02**	**0.65**
**SAM: Arousal**	**30**	**2.60 (1.59)**	**29**	**4.86 (1.94)**	−**4.91**	**57**	**< 0.01**	**1.26^∗^**
**SAM: Dominance**	**30**	**2.47 (1.66)**	**29**	**4.24 (2.21)**	−**3.49**	**57**	**< 0.01**	**0.90^∗^**
EDI: Drive for Thinness	29	14.38 (4.09)	24	15.96 (3.14)	–1.55	51	0.13	0.42
**EDI: Body dissatisfaction**	**27**	**16.81 (4.94)**	**25**	**20.92 (4.58)**	−**3.10**	**50**	**< 0.01**	**0.85**
EDI: Bulimia	29	15.00 (3.45)	26	16.69 (3.28)	–1.86	53	0.07	0.49
**LEAS-C Self**	**30**	**33.37 (6.22)**	**29**	**29.69 (5.61)**	**2.38**	**57**	**0.02**	**0.61**
BPQ: ANS	30	50.27 (11.83)	30	55.60 (15.66)	–1.49	58	0.14	0.38
BPQ: AW	30	100.93 (22.60)	30	103.63 (26.47)	–0.42	58	0.67	0.11
**BMI**	**30**	**21.00 (2.60)**	**30**	**16.98 (3.73)**	**4.85**	**58**	**< 0.01**	**1.24^∗^**
**SEBQ: Positive**	**30**	**59.80 (13.57)**	**26**	**44.62 (14.46)**	**4.05**	**54**	**< 0.01**	**1.07^∗^**
**SEBQ: Negative**	**30**	**48.93 (12.64)**	**26**	**59.69 (14.90)**	−**2.92**	**54**	**< 0.01**	**0.77**
ERQ: Reappraisal	30	28.90 (7.22)	30	28.73 (7.34)	0.09	58	0.93	0.02
ERQ: Suppression	30	12.73 (5.38)	30	13.50 (5.52)	–0.54	58	0.59	0.14

*Group assignment: Positive values of t indicate higher values in HC; negative values of t indicate higher values in AN.*

*HC, healthy controls; AN, patients with restrictive anorexia nervosa; SAM, Self-Assessment Manikin scale; EDI, Eating Disorder Inventory; LEAS-C Self, Level of Emotion Awareness for Children; BPQ, Body Perception Questionnaire; BMI, Body Mass Index; SEBQ, The Subjective Emotional Balance Questionnaire; ERQ, Emotion Regulation Questionnaire.*

*^a^p-values computed via bootstrapping based on 1,000 simulated random samples.*

*Differences significant at p < 0.05 without any correction are marked in bold type.*

*Differences labeled with an asterisk remained significant after applying the Bonferroni correction for inflated Type I error rate.*

### Relationship Between Increase in Skin Conductance and Psychological Variables

To test the associations between the self-report variables in our study and the physiological response to mirror exposure, we used the untransformed SC data to compute the% increase in SCL and SCR (1) from REST 1 to NEUTRAL (“Increase-neutral”) and (2) from REST 2 to MIRROR (“Increase-mirror”). The resulting values were approximately normally distributed in the case of SCL but were still severely positively skewed for SCR. We therefore also computed transformed variables using a logarithmic function [*y* = *ln(x* + *101*)] analogous to the one used with the original SCR variables. Correlation analyses were subsequently conducted on both transformed and untransformed data. In all cases, because of substantial deviations from normality, numerous outliers and limited sample size, *SE*s were computed using the bootstrapping method with 10,000 simulated samples.

Table 3 shows the descriptive statistics and correlations between all of the computed indexes of SC change between the resting and the stimulus-exposure conditions. The results show that changes in SC from REST 1 to NEUTRAL were almost unrelated to changes in SC from REST 2 to MIRROR.

**TABLE 3:** Correlation matrix of % increase in skin conductance parameters.

	*M (SD)*	*N*	Increase SCL—mirror	Increase SCL—neutral	Increase SCR—mirror	Increase SCR—neutral	Increase SCR—mirror trans.	Increase SCR—neutral trans.
Increase SCL—mirror	5.06 (10.57)	60	–					
Increase SCL—neutral	11.80 (23.45)	60	0.14	–				
Increase SCR—mirror	156.31 (377.80)	60	0.22**	–0.01	–			
Increase SCR—neutral	213.34 (508.57)	60	–0.10	0.44**	–0.01	–		
Increase SCR—mirror—transformed	4.94 (1.08)	60	0.34**	–0.04	0.79**	0.03	–	
Increase SCR—neutral—transformed	5.07 (1.25)	60	–0.24	0.44**	0.09	0.68**	0.03	–

**p < 0.01.

*Computed via bootstrapping based on 10,000 simulated random samples.*

Correlations between all psychological variables and BMI and the% increase in SC parameters are presented in Table 4. The values of correlation coefficients indicate that it was mainly the increase in SCR in the MIRROR condition that was associated with the psychological variables measured in the study. These relationships were generally stronger for the untransformed data; however, due to the large variance and severe skew in the SCR data, most of these weak to moderate correlations were non-significant when bootstrapping was used for significance testing.

**TABLE 4:** Correlations between psychological variables and the % increase in SC parameters in the NEUTRAL condition and the MIRROR condition (no Type I error correction applied).

		Increase in SCL			Increase in SCR		Increase in SCR—transformed	
	*M (SD)*	*N*	*Mirror*	*Neutral*	*Mirror*	*Neutral*	*Mirror*	*Neutral*
SAM: Valence	4.81 (1.56)	59	0.02	–0.10	0.45	–0.04	0.26	–0.09
SAM: Arousal	3.71 (2.09)	59	–0.07	–0.04	0.27	–0.06	0.10	–0.03
SAM: Dominance	3.34 (2.13)	59	–0.08	0.04	0.22	0.07	0.11	0.12
EDI: Drive for Thinness	15.09 (3.74)	53	0.14	0.02	–0.01	0.10	0.11	0.07
EDI: Body dissatisfaction	18.79 (5.16)	52	0.26	–0.08	0.22*	–0.22	0.12	–0.14
EDI: Bulimia	15.80 (3.45)	55	0.06	0.30	–0.00	0.30*	0.05	0.38*
LEAS-C Self	31.56 (6.16)	59	–0.12	−0.28*	0.00	−0.26*	0.03	–0.18
BPQ: ANS	52.93 (14.02)	60	0.29*	0.01	0.49*	–0.06	0.28	–0.08
BPQ: AW	102.28 (24.44)	60	0.20	0.03	0.31	–0.07	0.13	–0.15
BMI	18.99 (3.78)	60	–0.09	0.01	0.04	0.07	–0.04	0.14
SBEQ: Positive	52.75 (15.83)	56	0.01	0.01	−0.33*	0.02	–0.20	–0.06
SBEQ: Negative	53.93 (14.64)	56	0.06	0.07	0.31*	0.07	0.18	0.14
ERQ: Reappraisal	28.82 (7.22)	60	0.01	–0.16	–0.21	–0.02	–0.03	–0.18
ERQ: Suppression	13.12 (5.42)	60	0.18	0.13	0.26*	0.14	0.22	0.12

*SAM, Self-Assessment Manikin scale; EDI, Eating Disorder Inventory; LEAS-C Self, Level of Emotion Awareness for Children; BPQ, Body Perception Questionnaire; BMI, Body Mass Index; SEBQ, The Subjective Emotional Balance Questionnaire; ERQ, Emotion Regulation Questionnaire.*

*p < 0.05; **p < 0.01.

*Computed *via* bootstrapping based on 10,000 simulated random samples.*

The correlation analysis revealed the expected positive associations between SCR% increase in MIRROR and basic emotional dimensions indexed by SAM, especially the Valence dimension; however, these associations were not significant and, in the case of transformed data, weak. Significant positive associations were found between the untransformed increase in SCR in MIRROR and body dissatisfaction, self-reported autonomic nervous system reactivity (BPQ: ANS), frequency of negative emotions as measured by SBEQ, and suppression as an emotion regulation strategy (ERQ). A significant negative correlation was found with the frequency of positive emotions. The relationship between the increase in SCR in MIRROR and awareness of body processes (BPQ: AW) was also in the hypothesized direction, but non-significant. The relationships between the increase in SCR in MIRROR and BMI, drive for thinness, bulimia, emotional awareness as measured by LEAS-C, and reappraisal, were all non-significant and very weak. We also found somewhat surprising significant relationships between EDI Bulimia and LEAS-C Self scores and increase in SC parameters in NEUTRAL; however, given the high variance in SCR data and the fact that these relationships were not hypothesized, this finding could have been purely coincidental.

With respect to the BMI centile band, Spearman correlation analysis did not reveal significant correlations with any of the SC increase variables (all ρs < 0.21). Since the BMI centile band was strongly related to BMI (ρ = 0.94), the BMI centile band was omitted from the subsequent exploratory analysis.

Because the correlation analysis involved a number of psychological variables that were closely interrelated, some of the correlations presented in Table 4 were expected to be interdependent, and some might have been the result of the Type I error. Due to the interdependence, no correction was used to control for the Type I error; instead, to obtain a more general perspective on the factors underlying the correlation patterns, we conducted a supplementary principal component analysis (PCA) in order to explore the factorial structure of the data. First, we used Parallel Analysis (Horn, 1965) to determine the number of components for extraction. The Parallel Analysis indicated only two components with eigenvalues larger than those obtained through random permutations of the data. These components were subsequently extracted through PCA with Oblimin rotation. Variables loading primarily on Component 1, which could be labeled as the “Affective Sensitivity” component, included both subscales of SEBQ, both subscales of BPQ, all three SAM scores, ERQ Suppression, and the Body Dissatisfaction and—to a lesser degree—Drive for Thinness subscales of EDI. Higher scores on this component seemed to indicate higher sensitivity to negative affective stimuli and higher negative affect in general. Component 2, which could be loosely described as the “Adaptive Emotion Regulation” component, was primarily composed of variance in EDI Bulimia (high negative loading), LEAS-C Self, BMI, and the Reappraisal scale of ERQ. The two components were almost orthogonal (*r* = −0.03).

Subsequent correlation analysis with bootstrapped *SE*s confirmed that the increase in SCR in MIRROR was moderately positively related to Component 1 (*r* = 0.46, *p* < 0.05, and *r* = 0.30, ns, for untransformed and transformed increase in SCR, respectively), but was unrelated to Component 2 (*r*s = 0.01 and −0.06, ns). Independent-sample *t*-tests indicated the AN group (*n* = 23) scored significantly higher in Component 1 [*t*(48) = −3.33, *p* = 0.002, *g* = 0.93] and lower in Component 2 [*t*(48) = 2.54, *p* = 0.02, *g* = 0.71] than the HC group (*n* = 27).

### Supplementary *post hoc* Analysis: Interaction Between Diagnosis and Interoceptive Awareness

In general, our data indicated high variability within the AN group with respect to SC reactivity to mirror exposure, which was not paralleled in HCs. While the differences between the two groups in SCR were generally non-significant, instances of extreme changes in SCR in MIRROR were only observed in participants in the AN group. At the same time, the two groups differed significantly in some of the psychological variables that showed significant relationships with the increase in SCR during mirror exposure. This might have suggested an interaction between the presence vs. absence of diagnosis of restrictive anorexia and other characteristics independent of the diagnosis of anorexia, with only anorectic patients scoring high or low on this variable showing high physiological reactivity to mirror exposure.

**FIGURE 4:**
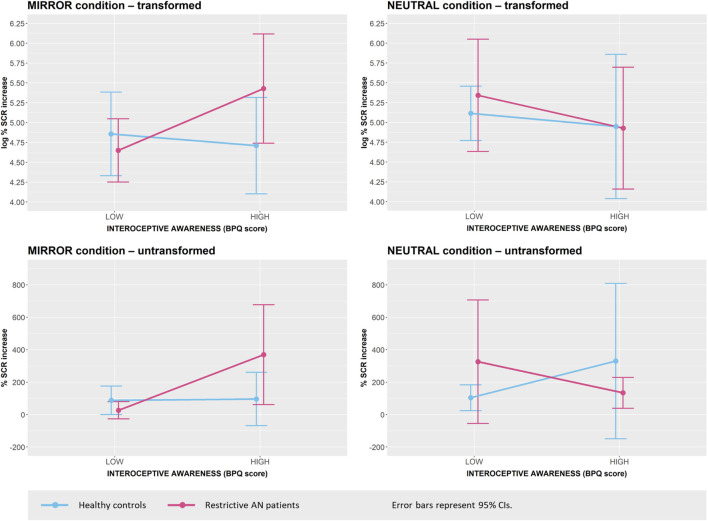
Increase in SCR during the MIRROR condition and the NEUTRAL condition by group and level of interoceptive awareness.

The present data showed that body awareness and autonomic nervous system reactivity measured by the BPQ correlated positively with the increase in SCR in MIRROR and loaded on the affective reactivity component but were not significantly related to the diagnosis of anorexia nervosa. To explore a potential interaction effect, we conducted an additional exploratory *post hoc* analysis of the relationships between the above-mentioned variables. First, we combined the strongly correlated (*r* = 0.77) BPQ: ANS and BPQ: AW scores into a single measure and performed a mean split, dividing the scores into two groups indicating high (*n* = 31) and low (*n* = 29) interoceptive awareness. We then performed two separate 2 × 2 factorial ANOVAs testing the effects of group (AN vs. HC) and interoceptive awareness (low vs. high) on the increase in SCR in MIRROR and in NEUTRAL. For the transformed data, there was an indicated interaction effect in the expected direction for MIRROR, but this effect was not significant [*F*(1, 56) = 2.84, *p* = 0.10, η_*p*_^2^ = 0.05]. Neither the main effect of group [*F*(1, 56) = 0.86, *p* = 0.36, η_*p*_^2^ = 0.02] nor the main effect of body awareness and reactivity [*F*(1, 56) = 1.48, *p* = 0.26, η_*p*_^2^ = 0.02] were significant. No similar interaction effect, or any main effect, were indicated for NEUTRAL (all *F*s < 1.0). The effects are visually displayed in Figure 4, which also contains the respective representations of the non-transformed data.

## Discussion

### Emotional Response to Mirror Exposure

The first objective of the present study was to analyze the differences in emotional response to mirror exposure between adolescent girls diagnosed with AN and matched healthy controls (HCs). Overall, our results support the hypothesis that adolescent girls with AN subjectively experience higher levels of negative affect and arousal and lower levels of control over their emotions during mirror exposure than girls without AN do. The first two observations are consistent with previous findings (e.g., Stice and Shaw, 2002; Kearney-Cooke and Tieger, 2015) as well as with common clinical experience. The feeling of reduced control over emotions in the AN group is a new finding in this area, as the dominance dimension is usually omitted in this kind of research; however, given the strong correlation between the dominance and valence scales of SAM (Bradley and Lang, 1994), this observation is not surprising.

### Physiological Response to Mirror Exposure

As expected, AN subjects showed lower levels of SCL compared to HCs across all experimental conditions. Similar to differences in subjective parameters, this generally reduced tonic SC is in line with previous findings (Vocks et al., 2007; Servián-Franco et al., 2015), indicating an overall reduced autonomic reactivity of AN patients, which can be explained by changes in sympathetic reactivity influenced by metabolic, emotional and cognitive factors typically associated with the restrictive type of AN (Palomba et al., 2017).

Regarding SCR, we expected that changes in physiological arousal would correspond to the experience of negative, highly arousing and less controllable emotions indicated by the psychological parameters; hence, AN patients were expected to show higher SCR in the experimental condition as compared to HCs. This hypothesis was not supported: Our study found no significant differences between the AN and the HC group across conditions. However, our results indicated substantial differences in variance in SCR (and, to a much lesser degree, SCL), especially in the exposure conditions. This finding is further discussed below in the context of other observations.

Interestingly, our study found no significant differences between the mirror exposure condition and the neutral exposure condition in either SCL or SCR across groups. The only differences observed were those between the resting conditions and the exposure conditions in general, with exposure to either the mirror or the neutral stimuli leading to a similar increase in SCL. This finding is quite surprising, and potential explanations should be offered with caution. The first possible explanation is a clinical one. In the HC group, a lack of difference in response to neutral geometric stimuli and body exposure *via* a mirror could simply result from mirror exposure having little effect beyond that attributable to increased alertness caused by the appearance of a new stimulus, since mirror exposure is not expected to represent such an aversive experience for healthy individuals as it does for highly body-dissatisfied individuals. This idea is in line with the fact that the HC group in our study indeed reported rather low levels of subjectively experienced arousal (*m* = 2.60 on a scale from 1 to 9) in response to mirror exposure. In the AN group, who reported significantly higher (though not extremely high) levels of arousal (*m* = 4.86), the lack of difference might be explained by the disruption of fundamental components of emotional processing in restrictive anorexia, leading to insufficient integration of signals from the body and emotional experience, associated with body image disturbances (see Zonnevylle-Bender et al., 2005; Nandrino et al., 2012). Although this explanation runs counter to our major hypothesis that patients with restrictive anorexia show stronger immediate physiological responses to mirror exposure due to a generally highly aversive emotional response, it seems plausible and is in line with our data. Similarly, it is possible that the participants employed some kind of avoidant behavior when briefly exposed to the mirror (e.g., lowering their eyes), which could have reduced their physiological reactivity. While the experimenter was checking throughout the experiment whether the girls were facing the presented stimuli, it could not be verified whether their visual attention was fully focused on the stimulus during exposure. Employment of various “covert” avoidance strategies of this kind might be an interesting area for further research with respect to body dissatisfaction and eating pathology.

Yet another possible explanation is a methodological one: In our study, geometric shapes in the shades of the gray (Jennings et al., 2007) were used as a neutral stimulus for the control exposure condition. The post-experimental debriefing revealed that this stimulus choice surprised some of the participants and raised suspicion that the first exposure condition would actually involve some kind of unexpected cognitive task, which they were unprepared for. It is therefore possible that the physiological response to the geometric stimulus was not actually analogous to physiological responses normally elicited by motivationally non-significant, emotionally neutral visual stimuli as intended. In the future, it might be advisable to select neutral stimuli from the International Affective Pictures System (Bradley and Lang, 2007) or a similar standardized set of images used for such purposes. Another possibility is to change the original instruction, “Please open your eyes and look at the picture,” to a more specific formulation that would neutralize any potential apprehension, such as: “Please open your eyes and look at the picture. You don’t have to remember or analyze the picture in any way—simply look at the objects on it.”

A combination of all of these explanations is also plausible. Importantly, it should be noted that, although SCR values were moderately to strongly correlated across conditions in the entire sample and the overall average magnitude of SCR did not differ between the two exposure conditions, the proportional increase in SCR from the preceding resting condition to the mirror exposure condition was virtually unrelated to the SCR increase in the “neutral” exposure condition, and the two variables also showed different patterns of correlations with the observed psychological variables, as discussed below. This indicates that the increase in SCR in the mirror exposure condition was at least partly determined by the systematic influence of factors other than those associated with the SCR increase in the “neutral” exposure condition.

### Differences in Affectivity, Emotion Regulation and Emotional and Interoceptive Awareness, and Relationships Between Psychological Variables and Electrodermal Activity

Subjective emotional state following mirror exposure, defined in terms of valence, arousal and controllability (dominance) of current emotions, was not significantly related to the increase in electrodermal activity. While the relationships were in the expected direction, they were too weak to approach significance. However, we did find significant positive associations between the untransformed increase in SCR during mirror exposure and long-term body dissatisfaction, ANS reactivity, emotional suppression, and frequency of negative emotional experiences, and a negative association with the frequency of positive emotional experiences. A positive relationship, albeit non-significant, was also detected between increase in SCR during mirror exposure and body awareness. These results are consistent with previous studies, which point to the depressive experience in patients with AN (Hughes, 2012; Ergüney Okumuş et al., 2019). However, while our intergroup comparisons indicated that AN patients were more dissatisfied with their own bodies and negative emotional experiences prevailed over positive ones to a significantly greater degree in their lives compared to healthy controls, we found no significant differences in either ANS reactivity or body awareness between the two groups. Moreover, the observed non-significant differences were in the direction opposite to the one that we hypothesized: AN patients showed an overall higher interoceptive sensitivity than healthy controls. Although this finding seems at odds with the overall rationale behind our hypothesis, a closer look at the data offers an immediate explanation: While interoceptive sensitivity is expected to represent an essential aspect or prerequisite of overall emotional awareness, it is also moderately to strongly associated with overall proneness to experience negative affect, which, in our study, was demonstrated by the fact that the BPQ scores loaded equally strongly on the “affective sensitivity” component yielded by the Principal Component Analysis as SEBQ and SAM scores, which are both indicators of negative affect levels. It is therefore plausible that the lack of significant difference in interoceptive awareness (BPQ scores) between the AN and the HC group in our data was due to the fact that a subgroup of restrictive anorectic patients experience substantially higher levels of negative affect, but at the same time exhibit muted psychophysiological activity and reactivity, as discussed above. This reasoning is in line with the results of our *post hoc* analysis, which indicated an interaction effect between the level of interoceptive awareness and the diagnosis of restrictive anorexia on the increase in SCR after mirror exposure. While the effect was not significant, which is unsurprising given the low power of the *post hoc* factorial ANOVA, the results indicated that it was AN patients with high interoceptive sensitivity who tended to respond particularly strongly to mirror exposure in terms of phasic electrodermal activity. This interaction might also explain the high variance in SCR data in the AN group as opposed to the HC group. Although we emphasize that this observation is merely preliminary and the result of a *post hoc*, low-power analysis, it provides another perspective on the previously proposed ideas on diminished emotional and interoceptive awareness in patients with restrictive anorexia (Pollatos et al., 2008; Eshkevari et al., 2014), and we believe it deserves more attention in future research, as it may help shed more light on the role of body awareness in development and treatment of this as well as other eating disorders. It might be the case, for example, that interoceptive awareness plays an important role in treatment responsiveness of AN patients (including responsiveness to mirror exposure therapy) or in the differentiation of AN patients in terms of symptoms that develop later in the progress of the illness (e.g., emergence of bulimic symptoms in patients with high sensitivity).

The hypothesis on diminished emotional awareness in restrictive anorexia is further supported by the observation that AN patients showed significantly lower scores in the performance-based LEAS-C Self compared to healthy controls, which, again, is in line with previous findings (Beadle et al., 2013). At the same time, it has to be mentioned that, when entered into PCA with the other individual-difference variables, emotional awareness did not load on the same component as BPQ scores and variables indexing emotional experience, and neither was it related to the SCR increase after mirror exposure.

Contrary to our hypotheses, no differences were found between anorexia patients and healthy controls in either suppression or reappraisal as emotion regulation strategies. There was, however, some indication of these strategies being associated with the increase in SCR after mirror exposure in the expected opposing directions—suppression was significantly positively related to the increase in SCR, while reappraisal was negatively, albeit non-significantly, related to the increase in SCR. These relationships were generally weak but are in line with previous findings on the effect of emotion regulation strategies on the physiological component of emotional experience (Gross, 1998) and deserve further attention in research regarding the role of emotion regulation strategies in the treatment of eating disorders.

### Limitations

A major limitation in our study was low power for detection of small to medium effects, especially with regard to correlation analysis. However, we also believe the small sample size could only be a part of the problem—it is possible that some aspect of the research design might have generally dampened the effect of mirror exposure on the outcome variables and hence reduced the proportion of informative variance in the SCR data. In line with this reasoning, neither the AN patients nor the healthy controls in our study reported, on average, particularly strong subjective emotional responses to the mirror exposure. Another indication of this problem is the apparent dependence of the observed effects involving SCR variables on the relatively sparse extreme values of SCR. The common practice in research involving SCR data is to use logarithmic transformation to achieve normally distributed data, which are subsequently entered into all analyses. In the present study, we chose instead to perform robust correlation analyses on both transformed and untransformed data, since the transformation resulted in equal weights being given to the negligible differences in sparse low values of the SCR variable of little information value and the large differences in sparse high values of SCR, which likely carried most of the information on individual differences in physiological reactivity to aversive stimuli. As a result, most correlations were substantially weaker after transformation was applied, and, although not negligible, could not reach significance in a sample of *N* = 60 or smaller. While we understand this potential explanation does not make the supportive evidence yielded by our study any stronger than the observed values by themselves provide, the fact that virtually all correlations were only significant when untransformed data were analyzed, and that these correlations were relatively substantial and in line with our hypotheses, does seem to suggest that it was only the variance in “outlier” values that indexed relevant increases in physiological reactivity in the mirror exposure condition. To tackle this potential issue in future research, larger samples need to be employed, and one has to make sure that the experimental mirror exposure situation is as analogous to a standard everyday situation of viewing oneself in a mirror as possible. Another way to increase the information value of this kind of research would be the employment of alternative control conditions. For example, a stressful cognitive control condition (e.g., arithmetic problems) would provide a measure of a more general stress reactivity and a reference criterion for the evaluation of reactivity to a clinically significant stimulus (i.e., body exposure).

Another potential limitation in the present study is the possibility that the HC group did not constitute a substantially representative “normal” control group and was more similar to the AN group in terms of ED symptoms than an average healthy girl of that age would be. Since participation in the experiment was completely voluntary, the girls who participated might have been interested in the study due to their own preoccupation with body image and weight issues. This would also explain why differences in the EDI Drive for Thinness scale were not significant between the two groups. In future research, it might be advisable to select participants for the control group directly on the basis of EDI scores. On the other hand, it has to be noted that preoccupation with body image and drive for thinness are, at least to a certain degree, common characteristics in adolescent girls and do not have to be necessarily associated with an imminent risk of developing an eating disorder, which is illustrated by the fact that although some of the HCs in our study might have shown elevated scores in some of the EDI subscales, none of the met the key diagnostic criteria for EDs. Another important question for further research is therefore also whether increased emotional response to body exposure *via* a mirror is mostly related to those aspects of EDs that are also found in the non-clinical population of girls and women, such as body dissatisfaction and low self-esteem, and whether the more unique clinical aspects of EDs like restrictive AN (e.g., specific emotion regulation strategies) might actually lead to reduced emotional reactivity, suppressing the effect of the other characteristics.

Finally, it should be noted that although our research was heavily inspired by previous studies on the effect of mirror exposure on EDA, several aspects of our design were unique, which might limit the comparability of the findings. Specifically, participants in our study were exposed to the mirror in a sitting (rather than standing) position to enhance EDA measurement accuracy, and the duration of stimulus exposure was much shorter compared to previous studies, which was in line with the main focus of our research, i.e., to study group differences in immediate emotional response to mirror exposure and its relationship to individual-difference variables, as opposed to monitoring the longitudinal effects of prolonged exposure. In addition, our participants were adolescent girls rather than adult women, which might have also partly affected the results, as discussed above, due to the specific role body concerns play at this developmental stage. On the other hand, we believe these unique features of our study make it more relevant in other respects, as research on mirror exposure in adolescent girls is scarce despite the fact that the ED diagnosis is frequently made at this age.

## Conclusion

The results of our study do not directly support the idea that brief and pure mirror exposure in patients with restrictive anorexia leads to significant changes in physiological reactivity compared to healthy individuals or neutral stimuli. However, our study does suggest that physiological reactivity during body exposure is associated with the overall level of negative emotional experiences and with interoceptive sensitivity, which are closely connected. While anorectic adolescent female patients generally show higher levels of negative affect and distress than healthy girls, their levels of interoceptive awareness might be diminished to different degrees, which might be the reason why, in our study, we observed a substantially larger variance in EDA reactivity in patients with restrictive anorexia than we observed in healthy controls. More research is needed regarding specific factors influencing interoceptive and emotional awareness in patients with anorexia, and the role all of these variables play in the underlying mechanism and effectiveness of body exposure therapy as a treatment for eating disorders, as well as in prognosis and treatment of AN patients in general.

## Data Availability Statement

The original contributions presented in the study are included in the article/Supplementary Material, further inquiries can be directed to the corresponding author/s.

## Ethics Statement

The studies involving human participants were reviewed and approved by Ethical Committee of The University Hospital Brno. Written informed consent to participate in this study was provided by the participants’ legal guardian/next of kin.

## Author Contributions

TeK designed and executed the study, prepared the experimental protocol, assisted with data analyses, and wrote a substantial part of the final manuscript. TM performed data analyses, finalized a part of the “Results” section, and revised the final manuscript. RR and JC pre-processed the electrodermal data for statistical analyses. MS, PL, and TeK co-wrote and co-edited the final manuscript. JN and PT performed the initial paedo-psychiatric examination of subjects before they entered the study. All authors contributed to the article and approved the submitted version.

## Conflict of Interest

The authors declare that the research was conducted in the absence of any commercial or financial relationships that could be construed as a potential conflict of interest.

## Publisher’s Note

All claims expressed in this article are solely those of the authors and do not necessarily represent those of their affiliated organizations, or those of the publisher, the editors and the reviewers. Any product that may be evaluated in this article, or claim that may be made by its manufacturer, is not guaranteed or endorsed by the publisher.
